# Epidemiology, treatment and outcomes of bloodstream infection due to vancomycin-resistant enterococci in cancer patients in a *vanB* endemic setting

**DOI:** 10.1186/s12879-020-04952-5

**Published:** 2020-03-18

**Authors:** Ouli Xie, Monica A. Slavin, Benjamin W. Teh, Ashish Bajel, Abby P. Douglas, Leon J. Worth

**Affiliations:** 1 Victorian Infectious Disease Service, Royal Melbourne Hospital, 300 Grattan St, Parkville, 3050 Australia; 2 Department of Infectious Diseases and Infection Prevention, Peter MacCallum Cancer Centre, Parkville, Australia; 3 National Centre for Infections in Cancer, Melbourne, Australia; 4 Department of Haematology, The Royal Melbourne Hospital, Parkville, Australia; 5 Department of Haematology, Peter MacCallum Cancer Centre, Parkville, Australia

**Keywords:** Vancomycin-resistant enterococci, Bacteremia, Leukemia, Teicoplanin, Linezolid

## Abstract

**Background:**

Vancomycin-resistant enterococcus (VRE) is an important cause of infection in immunocompromised populations. Few studies have described the characteristics of *vanB* VRE infection. We sought to describe the epidemiology, treatment and outcomes of VRE bloodstream infections (BSI) in a *vanB* predominant setting in malignant hematology and oncology patients.

**Methods:**

A retrospective review was performed at two large Australian centres and spanning a 6-year period (2008–2014). Evaluable outcomes were intensive care admission (ICU) within 48 h of BSI, all-cause mortality (7 and 30 days) and length of admission.

**Results:**

Overall, 106 BSI episodes were observed in 96 patients, predominantly *Enterococcus faecium vanB* (105/106, 99%). Antibiotics were administered for a median of 17 days prior to BSI, and 76/96 (79%) were neutropenic at BSI onset. Of patients screened before BSI onset, 49/72 (68%) were found to be colonised. Treatment included teicoplanin (59), linezolid (6), daptomycin (2) and sequential/multiple agents (21). Mortality at 30-days was 31%. On multivariable analysis, teicoplanin was not associated with mortality at 30 days.

**Conclusions:**

VRE BSI in a *vanB* endemic setting occurred in the context of substantive prior antibiotic use and was associated with high 30-day mortality. Targeted screening identified 68% to be colonised prior to BSI. Teicoplanin therapy was not associated with poorer outcomes and warrants further study for *vanB* VRE BSI in cancer populations.

## Background

Vancomycin-resistant enterococci (VRE) contribute significantly to the burden of healthcare-associated infections, particularly among immunocompromised patient populations [1–3]. Outcomes of infection include higher mortality [4–7] and prolonged hospitalisation [1, 8]. Notably, the proportion of vancomycin-resistant isolates has increased to almost 50% of *E. faecium* isolates in Australia in 2017 and over 20% of isolates in many European regions [9–11].

Vancomycin resistance mediated by *vanA* and *vanB* is due to inducible expression of peptidoglycan precursors terminating with d-Ala–d-Lac rather than d-Ala–d-Ala. This results in markedly lower affinity for binding of vancomycin [12]. In contrast to *vanA*, *vanB* is not strongly induced by teicoplanin and generally remains susceptible [13]. However, resistance may develop on teicoplanin therapy.

Studies evaluating risks for BSI and clinical outcomes have focussed on *vanA* VRE as the predominant genotype or have been performed in settings where *vanA* strains are dominant [2, 10]. Although there have been recent reports of increased *vanA* in Australia, *vanB* has historically been the predominant genotype [9]. Few studies describe risk factors and outcomes of *vanB* VRE BSI particularly in the high-risk hematology and oncology population. Two previous case-control studies have described risk factors for *vanB* VRE BSI in unselected patients [2, 14]. They described the use of central venous access devices (CVAD), neutropenia, allogeneic hematopoietic transplant, urinary catheterisation and duration of metronidazole therapy as risk factors. A study focusing on *vanB* VRE BSI in patients with hematological malignancy found acute myeloid leukemia and vancomycin therapy as risk factors [15]. A single case control study investigating factors influencing mortality and length of stay found prior intensive care unit stay and burden of comorbidities to be associated with mortality and linezolid therapy with lower mortality [1]. However, this study was not restricted to patients with malignancy and the median duration of neutropenia was only 1 day.

The objective of this study was to describe the epidemiology, treatment and outcomes of VRE BSI in patients with solid tumours or hematological malignancies in a *vanB* endemic setting at two large Australian healthcare facilities. We also sought to describe the prevalence of risk factors previously associated with *vanA* BSI in this population in order to inform targeted interventions for improved patient outcomes.

## Methods

### Setting

Retrospective chart review was performed at two adult tertiary hospitals in Melbourne, Australia: The Royal Melbourne Hospital (RMH) and the Peter MacCallum Cancer Centre (PMCC). The study period was defined as 1 January 2008 to 31 December 2013 (RMH) and 1 January 2008 to 30 June 2014 (PMCC). Both centres are major tertiary referral teaching hospitals with oncology and haematology units, including autologous (PMCC) and allogeneic (RMH) bone marrow transplant services. During this period, active surveillance for VRE was performed at both centres by collection of weekly perianal swabs from inpatients with no known history of VRE colonisation or infection. Patients who were VRE colonized on surveillance swabs or with a previous history of VRE in a clinical isolate were placed in contact precautions requiring the use gowns and gloves on entry to the patient room.

### Study population

Cases of vancomycin-resistant *E. faecium* or *E. faecalis* BSI during the defined study period were identified from laboratory extracts. Patients with underlying hematological or oncological diseases were eligible for inclusion. All patients with at least one positive blood culture isolating VRE were classified as having a BSI. Blood cultures were obtained with aseptic technique via peripheral venepuncture or through a CVAD.

### Laboratory confirmation

Chromogenic medium (ChromID VRE agar plate, Biomerieux) was used for detection and differentiation of VRE from screening swabs. Identification of *Enterococci* in bloodstream isolates was based on VITEK 2 (Biomerieux). Genetic testing for *vanA* and *vanB* were not routine during the study period. Clinical isolates were deemed to be *vanB* if phenotypically susceptible to teicoplanin and resistant to vancomycin by VITEK 2 (Biomerieux) using Clinical and Laboratory Standards Institute (CLSI) guidelines (CLSI 2015, version M100-S25). Isolates resistant to teicoplanin were confirmed to be *vanA* by PCR (Xpert *vanA*/*vanB*, Cepheid, Sunnyvale, USA).

### Data collection

Data including patient demographics, underlying disease, comorbidities, presence of CVAD or urinary catheters, WHO mucositis grade, treatment and outcomes were captured by retrospective chart review and using a standardised data collection tool. Consistent with previous studies, the Chronic Disease Score specific to VRE (CDS-VRE) was used as a quantitative measure of comorbidities for VRE infection [2, 16]. The number of days of antibiotic use in the 30 days preceding the first positive blood culture was obtained by medication chart review. The antibiotic used, sequence and duration of antibiotics were recorded. Evaluable outcomes included intensive care unit (ICU) admission within 48 h of first positive blood culture for VRE, all-cause mortality at 7 and 30 days, and length of hospital admission after BSI.

### Definitions

Days of neutropenia in the 30 days prior to first positive blood culture were defined as the number of days where the absolute neutrophil count was ≤0.5 × 10^9/L. Hypoalbuminemia was defined as a serum albumin of < 35 g/L. VRE colonization was defined as a positive rectal swab for VRE prior to first positive blood culture or previous VRE infection. Polymicrobial infection was defined as isolation of one or more additional organisms in a VRE-positive blood culture.

### Ethics review

The study was reviewed and approved by Human Research Ethics Committees at the Royal Melbourne Hospital (reference number: QA2015054) and Peter MacCallum Cancer Centre (reference number: 16/35R).

### Statistical analysis

Data analysis was performed using IBM SPSS Statistics version 26.0 (IBM Corp., Armonk, NY, USA). Categorical variables were expressed as frequencies. Continuous variables were reported as mean or median for normally or non-normally distributed data, respectively. Where a patient had more than one episode of VRE BSI, only the first episode was included in analysis. Univariable and multivariable logistic regression analyses were performed to evaluate the association between outcomes and potential risk factors, with covariates having *p* ≤ 0.2 included in multivariable models. To enable analysis of treatment outcomes, patients who only received teicoplanin, linezolid or daptomycin throughout the treatment course were grouped separately to patients receiving multiple or sequential antibiotics. Patients who were not treated due to end-of-life care were excluded from the logistic regression analyses.

## Results

### Patient characteristics

A total of 106 bloodstream infections in 96 patients were identified. Ten patients had more than one episode of BSI. Most patients had an underlying hematological malignancy (83/96, 86%), with the most common hematological malignancy being acute myeloid leukemia (40/83, 48%). There was no significant change in the number of cases per year through the study period. Patient characteristics are summarized in Table 1.

**Table 1:** Characteristics of patients with VRE bloodstream infection

**Characteristic**	
Male	50 (52%)
Age, median (years)	56 (range 20–78)
CDS-VRE score, median^†^	1.88 (range 0–4.97)
Hematological malignancy	83 (86%)
AML	40 (42%)
Allogeneic stem cell transplant	26 (27%)
On antibiotics prior to BSI^‡^	91/93 (98%)
Total antibiotic days prior to BSI, median^‡^	17 (range 0–65)
Hospital admissions prior to BSI, median^§^	1 (range 0–10)
Transfer from another hospital	20 (21%)
Days in hospital prior to BSI, median	16 (range 0–129)
ICU admission in 30 days prior to BSI	31 (32%)
In ICU at onset of BSI	19 (20%)
Neutropenic days prior to BSI, median	11 (range 0–30)
Neutropenic at time of BSI	76 (79%)
SIRS at time of BSI^†^	86/94 (91%)
Hypoalbuminemia days prior to BSI, median	17.5 (range 0–30)
Central venous access device^¶^	77/95 (81%)
Tunnelled	35/95 (37%)
Indwelling urinary catheter^†^	33/94 (35%)
Mucositis grade, median^‡^	0 (range 0–4)

*VRE* Vancomycin resistant enterococci, *CDS-VRE score* Chronic Disease Score specific to VRE, *AML* Acute myeloid leukaemia, *BSI* blood stream infection, *ICU* Intensive care unit, S*IRS* Systemic inflammatory response syndrome

^†^Data not available for 2 cases

^‡^Data not available for 3 cases

^¶^Data not available for 1 case

^§^12 months prior to VRE BSI

All isolates were *Enterococcus faecium* (Table 2). All cases demonstrated *vanB* phenotype except for one case of *vanA* VRE BSI during the study period. Twenty-three (24%) cases were polymicrobial BSI.

**Table 2:** Characteristics, treatment and outcomes of VRE bloodstream infections

**Characteristic**	
E. faecium	96 (100%)
*vanB*	95 (99%)
Patients with multiple episodes of VRE BSI	10 (10%)
Polymicrobial BSI	23 (24%)
Gram negative bacilli	9 (9%)
Candida sp.	4 (4%)
Coagulase negative staphylococci	10 (10%)
VRE screened prior to BSI	72 (75%)
Positive	49/72 (68%)
Treated with VRE active agent^a^	88/95 (93%)
Teicoplanin therapy alone	59/95 (62%)
Linezolid therapy alone	6/95 (6%)
Daptomycin therapy alone	2/95 (2%)
Sequential therapy	21/95 (22%)
ICU admission within 48 h of BSI	15 (16%)
Death at 7 days	8 (8%)
Death at 30 days	30 (31%)
Length of stay post BSI, median (days)	18 (range 1–78)
Length of stay total, median (days)	38.5 (range 2–138)

*VRE* Vancomycin resistant Enterococci, *BSI* Blood stream infection, *ICU* Intensive care unit

^a^Data not available for 1 patient

Antibiotics were administered for a median of 17 days prior to onset of VRE BSI. The most frequent antibiotics administered were piperacillin-tazobactam (68 patients, median duration 5 days, range 0–19), vancomycin (57 patients, median duration 2 days, range 0–30), and meropenem (52 patients, median duration 1 day, range 0–28). Most patients were neutropenic at the time of BSI (76/96, 79%) and had spent a median of 16 days (range 0–129 days) in hospital. A CVAD was present in most patients at the time of BSI (77/95, 81%), of which 35 were tunnelled.

Seventy two out of 96 patients were screened for VRE prior to BSI. Of these, forty-nine (68%) screened positive.

### Treatment and outcomes

Teicoplanin was used at any point during the treatment course in 80 patients (80/95, 84%) although 21 patients had sequential therapy with teicoplanin, daptomycin or linezolid. Teicoplanin was used alone throughout the treatment course in 59 patients, linezolid alone in 6 patients and daptomycin alone in 2 patients. Data regarding targeted antibiotic treatment were unavailable for 1 patient. Median duration of treatment in patients who received teicoplanin as a part of their treatment was 14 days (range 1–60). Teicoplanin was given as a 400-1200 mg load 12-hourly (6-12 mg/kg) for three doses then as 400-1200 mg daily maintenance with adjustment for renal function. No loading doses were given in 3 cases.

Seven patients were not treated (7/95, 7%). In four instances, patients were not treated as infection episodes were deemed to be line-related with prompt clinical improvement observed after line removal or were deemed to be contaminants by the treating physician. In three instances, patients were receiving end-of-life care and targeted therapy was therefore not commenced.

The majority of patients (86/94, 91%) met systemic inflammatory response syndrome (SIRS) criteria [17] at time of BSI onset. All-cause mortality was 8% at 7 days and 31% at 30 days. Median duration of hospital admission following BSI was 18 days (range 1–78 days). Intensive Care Unit (ICU) admission was required within 48 h for 15 patients (16%). Of the 10 patients with multiple episodes of VRE BSI, none were admitted to ICU or died within 7 days, and two died at 30 days.

### Risks for ICU admission and mortality

With respect to risk factors for ICU admission within 48 h of blood culture collection (Table 3), multivariable analysis demonstrated mucositis grade was independently associated with ICU admission (OR 1.81, 95% CI 1.04–3.14) and targeted treatment with teicoplanin was associated with lower odds of ICU admission (OR 0.14, 95% CI 0.03–0.66). Patients who only received linezolid or daptomycin therapy were not inputted into the regression model due to low numbers.

**Table 3:** Univariable and multivariable analysis of factors associated with ICU admission^a^ in patients with VRE bloodstream infection

	**ICU admission (*n = 13)***	**No ICU admission** **(*n* = 80)**	**Univariable odds ratio**	**95% CI**	***p*-value**	**Multivariable odds ratio**	**95% CI**	***p*-value**
**Age, median (IQR), years**	47 (40–63)	58 (43–66)	0.98	0.94–1.01	0.212			
**Male**	6 (46%)	43 (54%)	0.74	0.23–2.39	0.612			
**CDS-VRE, median (IQR)**	1.9 (1.5–3.6)	1.9 (1.8–3.4)	1.02	0.67–1.55	0.932			
**Hematological malignancy**	12 (92%)	69 (86%)	1.91	0.23–16.21	0.552			
**AlloBMT**	6 (46%)	18 (23%)	2.95	0.88–9.90	0.080	0.96	0.16–5.96	0.968
**Antibiotic days prior to BSI, median (IQR)**	20 (18–29)	16 (9–26)	1.03	0.99–1.08	0.163	1.02	0.97–1.08	0.399
**ICU admission**^b^	4 (31%)	25 (31%)	0.98	0.28–3.48	0.972			
**Hypoalbuminemia days**^b^**, median (IQR)**	18 (14–30)	17 (9–29)	1.03	0.97–1.09	0.380			
**Mucositis grade, median (IQR)**	3 (0–4)	0 (0–2)	1.60	1.11–2.30	0.011	1.81	1.04–3.14	**0.036**
**Polymicrobial BSI**	3 (23%)	18 (23%)	1.03	0.26–4.16	0.963			
**Neutropenia at time of BSI**	9 (69%)	66 (83%)	0.48	0.13–1.77	0.269			
**Teicoplanin monotherapy**	4 (31%)	55 (69%)	0.23	0.06–0.83	0.024	0.14	0.03–0.66	**0.013**

*CDS-VRE score* Chronic Disease Score specific to VRE, *CKD* chronic kidney disease, *AML* Acute myeloid leukemia, *alloBMT* allogeneic bone marrow transplant

^a^ICU admission within 48-h of bloodstream infection

^b^In 30 days prior to bloodstream infection

With respect to risk factors for 30-day mortality (Table 4), multivariable analysis revealed ICU admission within 48 h of BSI to be associated with higher odds of 30-day mortality (OR 4.16, 95% CI 1.08–16.00). Teicoplanin monotherapy throughout the treatment course was not associated with 30-day mortality (OR 0.57, 95% CI 0.20–1.64).

**Table 4:** Univariable and multivariable analysis of factors associated with 30-day all-cause mortality following VRE bloodstream infection

	**Patients who died (*n* = 27)**	**Patients who survived (*n* = 66)**	**Univariable odds ratio**	**95% CI**	***p*-value**	**Multivariable odds ratio**	**95% CI**	***p*-value**
**Age, median (IQR), years**	55 (44–65)	58 (43–66)	1.00	0.97–1.03	0.964			
**Male**	15 (56%)	34 (52%)	1.18	0.48–2.89	0.723			
**CDS-VRE, median (IQR)**	1.9 (1.9–3.9)	1.9 (1.5–3.4)	1.40	0.99–1.96	0.055	1.38	0.96–1.99	0.082
**Haematological malignancy**	25 (93%)	56 (85%)	2.23	0.46–10.94	0.322			
**AlloBMT**	9 (33%)	15 (23%)	1.70	0.63–4.56	0.291			
**Hypoalbuminemia days**^†^**, median (IQR)**	22 (11–30)	16 (10–23)	1.05	1.00–1.10	0.058	1.04	0.98–1.09	0.186
**Mucositis grade, median (IQR)**	0 (0–3)	0 (0–2)	1.15	0.86–1.54	0.336			
**Polymicrobial BSI**	7 (26%)	14 (21%)	1.30	0.46–3.69	0.622			
**Neutropenia at time of BSI**	20 (74%)	55 (83%)	0.57	0.20–1.68	0.308			
**Teicoplanin monotherapy**	14 (52%)	45 (68%)	0.54	0.22–1.38	0.200	0.57	0.20–1.64	0.299
**ICU 48 h after BSI**	8 (30%)	5 (8%)	5.14	1.50–17.58	0.009	4.16	1.08–16.00	**0.038**

*CDS-VRE score* Chronic Disease Score specific to VRE, *CKD* Chronic kidney disease, *AML* Acute myeloid leukaemia, *alloBMT* allogeneic bone marrow transplant, *BSI* Blood stream infection

^†^In 30 days prior to bloodstream infection

## Discussion

Our study describes the characteristics and outcomes of predominantly *vanB*-phenotype VRE BSI in a tertiary hematology and oncology setting. The most common underlying condition in this group of patients was acute myeloid leukemia (AML). We found substantive antibiotic use prior to onset of BSI and high 30-day mortality after VRE BSI similar to that described for *vanA* VRE [18–20]. We observed targeted treatment with teicoplanin to not be associated with 30-day mortality. Colonization with VRE was identified by screening prior to the onset of BSI in a large proportion (68%) of patients, supporting early commencement of VRE-active empiric therapy as a component of appropriate sepsis management in patients with known colonization.

Previous studies including predominantly *vanA* isolates have identified prior antibiotic exposure, hypoalbuminemia, neutropenia and the presence of indwelling venous catheters to be risk factors for development of BSI [14, 15, 18, 21–23]. Our study found a median of 11 days of hypoalbuminemia and 11 days of neutropenia prior to onset of BSI. Prior studies have demonstrated a median of 13 days of hypoalbuminemia and between 1 and 32 days of neutropenia with longer durations of neutropenia in allogeneic hematopoietic transplant patients [2, 14, 18]. Most patients in our study (81%) had a CVAD at the time of BSI. AML and exposure to vancomycin have been associated with *vanB* VRE bacteraemia previously [15]. Consistent with these findings, we found AML as the most frequent underlying condition. In addition, we found a median of 17 antibiotic days exposure prior to BSI, generally related to administration of broad-spectrum agents (meropenem, piperacillin-tazobactam or vancomycin). While the cumulative duration of antibiotic exposure was not reported, the median duration of exposure to vancomycin, piperacillin-tazobactam and meropenem is similar to that reported previously [1, 21].

We found death at 30 days was 31% and comparable to previous Australian studies of *vanB* VRE BSI between 21 and 36% [1, 15]. Mortality after VRE BSI among patients with hematological malignancies was also similar to previous reports [18–20]. Few previous studies have investigated treatment outcomes associated with teicoplanin for *vanB* VRE BSI. A case-control study in Australia suggested that linezolid treatment for *vanB* VRE was associated with lower in-hospital mortality than teicoplanin [1]. We found that treatment with teicoplanin monotherapy was associated with lower rates of ICU admission within 48 h of VRE BSI. However, this may have been influenced by the selective use of linezolid or daptomycin in more unwell patients, rather than an effect of teicoplanin itself. Comparison is also limited by the smaller number of patients receiving linezolid and daptomycin therapy. Targeted treatment with teicoplanin was not associated with increased mortality at 30 days.

We identified mucositis to be associated with need for ICU admission within 48 h of VRE BSI. This may reflect a greater disturbance of innate immunological barriers predisposing to infection. There was a concurrent high rate of polymicrobial BSI in our cohort consisting of almost a quarter of all episodes. However, mucositis and polymicrobial BSI were not associated with higher 30-day mortality.

Others have proposed that VRE BSI may contribute to mortality or may just be a marker for severe underlying disease with conflicting reports on the effectiveness of early empiric therapy [1, 4, 24–27]. It has been reported that in patients with hematological malignancy, rates of severe sepsis within 2 days of VRE BSI can be as high as 36% [6]. Sixteen percent of our patients required ICU admission within 48 h of onset of BSI and the need for ICU admission was associated with 30-day mortality on multivariable analysis, although the confidence interval was wide (OR 4.16, 95% CI 1.08–16.00). Septic shock at onset of BSI has previously been associated with mortality at 28-days with a hazard ratio of 1.91 [26].

The median length of hospitalization after VRE BSI was 18 days. This was the comparable to previous studies for *vanB* VRE BSI and for malignant hematology patients in a *vanA* endemic setting [1, 20].

Rectal colonization with VRE has been reported as a risk factor for subsequent BSI in recipients of allogeneic hematopoietic stem cell transplantation [4, 28, 29]. Notably, 68% of our cohort who were screened for VRE prior to BSI were positive, highlighting the potential benefits of screening to guide empiric therapy. We suggest that while screening for colonization by collection of rectal swabs will not identify all patients who develop VRE BSI, when combined with other clinical risk factors such as mucositis and prior antibiotic exposure, it can help identify patients at high risk. Risk scores, such as that developed by Webb et al. may aid risk stratification [28], which may then inform treatment decisions (e.g. administration of VRE-active antimicrobial agents) in high-risk, and unwell patients. Another approach could be the early addition of VRE-active antibiotics in patients known to be VRE colonized who develop positive blood cultures with gram-positive cocci resembling streptococci/enterococci. Protocols for neutropenic sepsis at our institutions have changed over recent years to reflect the latter approach.

A limitation of this study is the retrospective design, and inability to confirm causal association between risk factors and outcomes. Genotyping for *vanA*/*vanB* genes were not performed as routine during the study period. VRE isolates with teicoplanin-susceptible phenotype but harbouring the *vanA* gene have been described. However, Australia-wide national surveillance had described low rates of *vanA* during the study period and undetected *vanA* isolates were unlikely to have contributed significantly to the results [30].

## Conclusion

In summary, we report clinical characteristics and outcomes of a large cohort of patients with *vanB* VRE bacteremia within a hematology and oncology setting. VRE BSI in a *vanB* predominant environment occurred in the context of significant antibiotic use and was associated with high 30-day mortality similar to that described for *vanA* BSI. We did not find that teicoplanin was associated with poorer outcomes and further comparative study of teicoplanin as an option for treatment in *vanB* VRE BSI is warranted. Screening for VRE carriage with rectal swabs identified more than two thirds of patients with subsequent VRE BSI, highlighting the benefit of surveillance for informing decisions regarding early effective treatment in the setting of sepsis within this vulnerable population.

## Data Availability

The datasets used and/or analysed during the current study are available from the corresponding author on reasonable request.

## Abbreviations

AML: Acute myeloid leukemia
BSI: Bloodstream infection
CVAD: Central venous access device
ICU: Intensive care unit
PMCC: Peter MacCallum Cancer Centre
RMH: The Royal Melbourne Hospital
VRE: Vancomycin resistant enterococci

## References

[1] Cheah AL, Spelman T, Liew D, Peel T, Howden BP, Spelman D (2013). Enterococcal bacteraemia: factors influencing mortality, length of stay and costs of hospitalization. Clin Microbiol Infect.

[2] Cheah AL, Peel T, Howden BP, Spelman D, Grayson ML, Nation RL (2014). Case-case-control study on factors associated with vanB vancomycin-resistant and vancomycin-susceptible enterococcal bacteraemia. BMC Infect Dis.

[3] Shay DK, Maloney SA, Montecalvo M, Banerjee S, Wormser GP, Arduino MJ (1995). Epidemiology and mortality risk of vancomycin-resistant enterococcal bloodstream infections. J Infect Dis.

[4] Vydra J, Shanley RM, George I, Ustun C, Smith AR, Weisdorf DJ (2012). Enterococcal bacteremia is associated with increased risk of mortality in recipients of allogeneic hematopoietic stem cell transplantation. Clin Infect Dis.

[5] Hayakawa K, Marchaim D, Martin ET, Tiwari N, Yousuf A, Sunkara B (2012). Comparison of the clinical characteristics and outcomes associated with vancomycin-resistant Enterococcus faecalis and vancomycin-resistant E. faecium bacteremia. Antimicrob Agents Chemother.

[6] Satlin MJ, Soave R, Racanelli AC, Shore TB, van Besien K, Jenkins SG (2014). The emergence of vancomycin-resistant enterococcal bacteremia in hematopoietic stem cell transplant recipients. Leuk Lymphoma.

[7] DiazGranados CA, Zimmer SM, Klein M, Jernigan JA (2005). Comparison of mortality associated with vancomycin-resistant and vancomycin-susceptible enterococcal bloodstream infections: a meta-analysis. Clin Infect Dis.

[8] Butler AM, Olsen MA, Merz LR, Guth RM, Woeltje KF, Camins BC (2010). Attributable costs of enterococcal bloodstream infections in a nonsurgical hospital cohort. Infect Control Hosp Epidemiol.

[9] AURA (2019). 2019: third Australian report on antimicrobial use and resistance in human health.

[10] Mendes RE, Castanheira M, Farrell DJ, Flamm RK, Sader HS, Jones RN (2016). Longitudinal (2001-14) analysis of enterococci and VRE causing invasive infections in European and US hospitals, including a contemporary (2010-13) analysis of oritavancin in vitro potency. J Antimicrob Chemother.

[11] European Centre for Disease Prevention and Control. Surveillance of antimicrobial resistance in Europe 2016. Annual report of the European antimicrobial resistance surveillance network (EARS-net). Stockholm: ECDC; 2017.

[12] Faron ML, Ledeboer NA, Buchan BW (2016). Resistance mechanisms, epidemiology, and approaches to screening for Vancomycin-resistant Enterococcus in the health care setting. J Clin Microbiol.

[13] Cetinkaya Y, Falk P, Mayhall CG (2000). Vancomycin-resistant enterococci. Clin Microbiol Rev.

[14] Peel T, Cheng AC, Spelman T, Huysmans M, Spelman D (2012). Differing risk factors for vancomycin-resistant and vancomycin-sensitive enterococcal bacteraemia. Clin Microbiol Infect.

[15] Worth LJ, Thursky KA, Seymour JF, Slavin MA (2007). Vancomycin-resistant Enterococcus faecium infection in patients with hematologic malignancy: patients with acute myeloid leukemia are at high-risk. Eur J Haematol.

[16] McGregor JC, Perencevich EN, Furuno JP, Langenberg P, Flannery K, Zhu J (2006). Comorbidity risk-adjustment measures were developed and validated for studies of antibiotic-resistant infections. J Clin Epidemiol.

[17] Bone RC, Balk RA, Cerra FB, Dellinger RP, Fein AM, Knaus WA (1992). Definitions for sepsis and organ failure and guidelines for the use of innovative therapies in sepsis. The ACCP/SCCM consensus conference committee. American College of Chest Physicians/Society of Critical Care Medicine. Chest..

[18] Kang Y, Vicente M, Parsad S, Brielmeier B, Pisano J, Landon E (2013). Evaluation of risk factors for vancomycin-resistant Enterococcus bacteremia among previously colonized hematopoietic stem cell transplant patients. Transpl Infect Dis.

[19] Cho SY, Lee DG, Choi SM, Kwon JC, Kim SH, Choi JK (2013). Impact of vancomycin resistance on mortality in neutropenic patients with enterococcal bloodstream infection: a retrospective study. BMC Infect Dis.

[20] Kraft S, Mackler E, Schlickman P, Welch K, DePestel DD (2011). Outcomes of therapy: vancomycin-resistant enterococcal bacteremia in hematology and bone marrow transplant patients. Support Care Cancer.

[21] Gouliouris T, Warne B, Cartwright EJP, Bedford L, Weerasuriya CK, Raven KE (2018). Duration of exposure to multiple antibiotics is associated with increased risk of VRE bacteraemia: a nested case-control study. J Antimicrob Chemother.

[22] Husni R, Hachem R, Hanna H, Raad I (2002). Risk factors for vancomycin-resistant Enterococcus (VRE) infection in colonized patients with cancer. Infect Control Hosp Epidemiol.

[23] McKinnell JA, Kunz DF, Chamot E, Patel M, Shirley RM, Moser SA (2012). Association between vancomycin-resistant enterococci bacteremia and ceftriaxone usage. Infect Control Hosp Epidemiol.

[24] Tavadze M, Rybicki L, Mossad S, Avery R, Yurch M, Pohlman B (2014). Risk factors for vancomycin-resistant enterococcus bacteremia and its influence on survival after allogeneic hematopoietic cell transplantation. Bone Marrow Transplant.

[25] Bae KS, Shin JA, Kim SK (2018). Han SB.

[26] Han SH, Chin BS, Lee HS, Jeong SJ, Choi HK, Kim CO (2009). Vancomycin-resistant enterococci bacteremia: risk factors for mortality and influence of antimicrobial therapy on clinical outcome. J Inf Secur.

[27] Kamboj M (2018). Cohen N.

[28] Webb BJ, Healy R, Majers J, Burr Z, Gazdik M, Lopansri B (2017). Prediction of bloodstream infection due to Vancomycin-resistant Enterococcus in patients undergoing leukemia induction or hematopoietic stem-cell transplantation. Clin Infect Dis.

[29] MacAllister TJ, Stohs E, Liu C, Bryan A, Whimbey E, Phipps A (2018). 10-year trends in vancomycin-resistant enterococci among allogeneic hematopoietic cell transplant recipients. J Inf Secur.

[30] AURA (2016). 2016: first Australian report on antimicrobial use and resistance in human health.

